# Stratifying patients with peripheral neuropathic pain based on sensory profiles: algorithm and sample size recommendations

**DOI:** 10.1097/j.pain.0000000000000935

**Published:** 2017-05-02

**Authors:** Jan Vollert, Christoph Maier, Nadine Attal, David L.H. Bennett, Didier Bouhassira, Elena K. Enax-Krumova, Nanna B. Finnerup, Rainer Freynhagen, Janne Gierthmühlen, Maija Haanpää, Per Hansson, Philipp Hüllemann, Troels S. Jensen, Walter Magerl, Juan D. Ramirez, Andrew S.C. Rice, Sigrid Schuh-Hofer, Märta Segerdahl, Jordi Serra, Pallai R. Shillo, Soeren Sindrup, Solomon Tesfaye, Andreas C. Themistocleous, Thomas R. Tölle, Rolf-Detlef Treede, Ralf Baron

**Affiliations:** a Department of Pain Medicine, BG University Hospital Bergmannsheil GmbH, Ruhr-University Bochum, Bochum, Germany; b Center of Biomedicine and Medical Technology Mannheim CBTM, Medical Faculty Mannheim, Heidelberg University, Heidelberg, Germany; c INSERM U-987, Centre d'Evaluation et de Traitement de la Douleur, CHU Ambroise Paré, Boulogne-Billancourt, France; d Université Versailles-Saint-Quentin, Versailles, France; e Nuffield Department of Clinical Neurosciences, University of Oxford, Oxford, United Kingdom; f Department of Neurology, BG University Hospital Bergmannsheil GmbH, Ruhr-University Bochum, Bochum, Germany; g Department of Neurology, Danish Pain Research Center, Aarhus University Hospital, Aarhus, Denmark; h Department of Anaesthesiology, Critical Care Medicine, Pain Therapy and Palliative Care, Pain Center Lake Starnberg, Benedictus Hospital Tutzing, Tutzing, Germany; i Anaesthesiological clinic, Klinikum rechts der Isar, Technische Universität München, Munich, Germany; j Division of Neurological Pain Research and Therapy, Department of Neurology, Universitätsklinikum Schleswig-Holstein, Campus Kiel, Kiel, Germany; k Departments of Helsinki University Central Hospital, Helsinki, Finland; l Etera Mutual Pension Insurance Company, Helsinki, Finland; m Department of Pain Management and Research, Division of Emergencies and Critical Care, Oslo University Hospital, Oslo, Norway; n Department of Molecular Medicine and Surgery, Karolinska Institute, Stockholm, Sweden; o Pain Research, Department of Surgery and Cancer, Imperial College, London, United Kingdom; p H. Lundbeck A/S, Copenhagen, Denmark; q Department of Physiology and Pharmacology, Karolinska Institute, Stockholm, Sweden; r Neuroscience Technologies, Ltd, Barcelona, Spain; s Diabetes Research Unit, Sheffield Teaching Hospitals NHS Foundation Trust, Sheffield, United Kingdom; t Brain Function Research Group, School of Physiology, Faculty of Health Sciences, University of the Witwatersrand, Johannesburg, South Africa; u Department of Neurology, Odense University Hospital, Odense, Denmark; v Department of Neurology, Klinikum rechts der Isar, Technische Universität München, Munich, Germany

**Keywords:** Quantitative sensory testing, German Research Network on Neuropathic Pain, Diabetic polyneuropathy, Peripheral nerve injury, Postherpetic neuralgia

## Abstract

In a recent cluster analysis, it has been shown that patients with peripheral neuropathic pain can be grouped into 3 sensory phenotypes based on quantitative sensory testing profiles, which are mainly characterized by either sensory loss, intact sensory function and mild thermal hyperalgesia and/or allodynia, or loss of thermal detection and mild mechanical hyperalgesia and/or allodynia. Here, we present an algorithm for allocation of individual patients to these subgroups. The algorithm is nondeterministic—ie, a patient can be sorted to more than one phenotype—and can separate patients with neuropathic pain from healthy subjects (sensitivity: 78%, specificity: 94%). We evaluated the frequency of each phenotype in a population of patients with painful diabetic polyneuropathy (n = 151), painful peripheral nerve injury (n = 335), and postherpetic neuralgia (n = 97) and propose sample sizes of study populations that need to be screened to reach a subpopulation large enough to conduct a phenotype-stratified study. The most common phenotype in diabetic polyneuropathy was sensory loss (83%), followed by mechanical hyperalgesia (75%) and thermal hyperalgesia (34%, note that percentages are overlapping and not additive). In peripheral nerve injury, frequencies were 37%, 59%, and 50%, and in postherpetic neuralgia, frequencies were 31%, 63%, and 46%. For parallel study design, either the estimated effect size of the treatment needs to be high (>0.7) or only phenotypes that are frequent in the clinical entity under study can realistically be performed. For crossover design, populations under 200 patients screened are sufficient for all phenotypes and clinical entities with a minimum estimated treatment effect size of 0.5.

## Abstract 2

Supplemental Digital Content is Available in the Text.

Phenotype stratification of patients with peripheral neuropathic pain can be conducted with a novel algorithm based on sensory profiles.

## 1. Introduction

Neuropathic pain is defined as pain as a result of a lesion or disease of the somatosensory nervous system^18,37^ and may involve diverse etiologies including diabetes, HIV, chemotherapy, herpes zoster, or nerve injury. Historically, neuropathic pain is classified based on these etiologies, although similar symptoms and signs are frequent across these etiologies. Furthermore, it has become evident in the last decades that an etiology-based classification approach of patients is not sufficient, as first-line treatment is often inefficient in more than half of the patients.^17^ At the same time, a number of promising new drugs have failed late trial stages.^17,23^ Better patient stratification might improve clinical trial outcome.

Although all neuropathic pain states result from a lesion or disease of the somatosensory nervous system, the pathogenesis and subsequently pathophysiological mechanisms in damaged and surviving afferent nerve fibers such as conduction block, ectopic impulse generation, peripheral and central sensitization may differ between patients. Although these mechanisms often cannot be tested in patients directly, the individual patient's sensory profile, including sensory signs such as hyperalgesia, allodynia, or hypoesthesia may be linked to mechanisms.^4^ A comprehensive way of assessing the sensory profile of a patient is quantitative sensory testing (QST) in accordance with the protocol of the German Research Network on Neuropathic Pain (DFNS).^3,33^

In a recent study,^5^ we have shown that QST profiles of patients with peripheral neuropathic pain reveal 3 distinct phenotypes, that are (mainly) characterized by (1) thermal and mechanical sensory loss (SL) (referred to as “sensory loss” subsequently), (2) preserved sensory function, associated with mild heat or cold hyperalgesia (labeled “thermal hyperalgesia” subsequently), and (3) loss of thermal sensation, combined with mechanical hyperalgesia (MH) or allodynia (referred to as “mechanical hyperalgesia” subsequently). These phenotypes can be found across etiologies, but vary in frequency between these, which has been validated in 2 independent patient cohorts. To use these phenotypes to stratify patients in clinical trials or to suggest an efficient treatment for a patient, a standardized individual allocation of patients must be available.

The aim of this article is to provide an algorithm based on the results of our previous work^5^ and to estimate probabilities for individual patients to be allocated to each of the 3 named phenotypes. Based on the patients recruited and published by the consortia DFNS (German Research Network on Neuropathic Pain),^29^ IMI (Innovative Medicines Initiative) Europain,^10^ Neuropain,^5^ and Pain in Neuropathy Study (PiNS),^36^ for neuropathic pain due to diabetic polyneuropathy, peripheral nerve injury, and after herpes zoster, we provide frequencies of phenotypes and suggest that minimum sample sizes for phenotype-stratified trials.

## 2. Methods

### 2.1. Consortia

The international consortia DFNS (German Research Network on Neuropathic Pain^29^), IMI (Innovative Medicines Initiative) Europain, Neuropain,^5,10^ and Pain in Neuropathy Study (PiNS^36^) participated in collecting and analyzing these data. All participating centers underwent strict quality control,^28,40^ and a recent analysis of heterogeneity between centers has shown that the data can be analyzed as a homogenous data set.^39^

### 2.2. Quantitative sensory testing protocol

Quantitative sensory testing according to the DFNS protocol assesses 13 parameters: cold detection threshold and warm detection threshold (WDT), thermal sensory limen, paradoxical heat sensations (PHS), cold pain and heat pain thresholds, mechanical pain threshold and mechanical pain sensitivity (MPS), dynamical mechanical allodynia (DMA), pressure pain threshold (PPT), wind-up ratio, tactile (mechanical) detection threshold, and vibration detection threshold. Thermal sensory and pain thresholds were measured using either a TSA 2001-II (MEDOC, Israel) or an MSA (SOMEDIC, Sweden) that increased or decreased temperature by 1°C per second.^33,34^ Mechanical detection threshold was defined as the geometric mean of 5 series of stimuli ascending and descending between 0.25 and 512 mN by a standardized set of von Frey hairs, mechanical pain threshold as the geometric mean of 5 series of stimuli ascending and descending by applying pinprick stimuli between 8 and 512 mN with a standardized pinprick set (MRC systems, Heidelburg, Germany).^34^ Mechanical pain sensitivity and DMA were assessed by applying a total of 50 stimuli (35 pinprick and 15 light tactile in a balanced protocol) and asking patients to give a pain rating on a 0 (no pain) to 100 (most intense pain imaginable) Numerical Rating Scale (NRS) scale. Mechanical pain sensitivity was calculated as the geometric mean of the pain ratings of the pinprick stimuli and DMA as the geometric mean of the pain rating of the tactile stimuli. For the wind-up ratio, the perceived intensity of a single pinprick stimulus was compared with that of a series of 10 repetitive pinprick stimuli of the same physical intensity on a 0 to 100 NRS scale, as an average of 5 series.^34^ Vibration detection threshold was assessed with a Rydel–Seiffer-graded tuning fork (64 Hz, 8/8 scale, mean of 3 testing series), and PPT was determined over muscle with a pressure gauge device (FDN200; Wagner Instruments, Greenwich, CT), exerting forces up to 2000 kPa, as a mean of 3 series of ascending stimulus intensities, each slowly increasing (50 kPa/s).^34^

### 2.3. Z-transformation

The initial assessment of 180 healthy subjects from the DFNS reference database revealed that all parameters except PHS and DMA could be transformed (partly in log-space) to a standard normal distribution.^28,31,33^ This process, called Z-transformation, normalizes all values to a mean = 0 and an SD = 1. Subsequently, all QST results of patients and healthy subjects were transformed in accordance with this normalization. Abnormal values are defined as values beyond the 95% confidence interval (CI). On a *z*-scale, this is represented by *z*-values <−1.96 or >1.96. Paradoxical heat sensation and DMA usually do not appear in healthy subjects and therefore cannot be transferred to the same scale. Thus, PHS was transformed to a binary 0/2-variable showing absence (coded as 0) or presence (coded as +2) of pathological values. Dynamical mechanical allodynia was transformed to a 0/2/3-variable representing no DMA (coded as 0), DMA with average pain ratings below 1 (coded as +2), and DMA with average pain ratings between 1 and 100 (coded as +3). The z-transformation normalizes for age decade, sex, and tested body region, thus making pain and detection thresholds comparable between patients with different age and sex and independently of the affected area, ie, nerves affected eg, at the face or the feet.

### 2.4. Sorting algorithm and validation

The most common way to establish a sorting algorithm would be to calculate the distance of the QST profile of an individual patient to each cluster centroid. This would, however, exclude all patients with one or more missing QST values (unless these would be filled in with imputed values), as the distance to a missing value cannot be calculated. As single missing values are not uncommon in patients with neuropathic pain and imputations could bias the results, we decided to use an approach with more intuitive handling of missing values. Because QST *z*-values are approximately normally distributed, our approach was based on normally distributed probabilities. For each QST *z*-value of each parameter *i* and patient *n*, a probability can be calculated for a phenotype to be present based on the density function of the said phenotype:
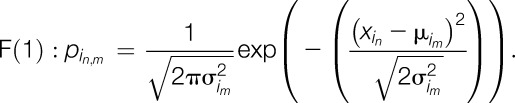
with *i* = one of 13 QST parameters, *n* = the *n*th patient in a set of patients, *m* = one of 3 phenotypes and conclusively, 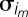 being the SD of the *i*th QST parameter for the *m*th phenotype in the defining data set,^5^ 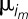 being the mean *z*-value of the same QST parameter and phenotype in the defining data set,^5^ and finally 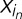 being the *z*-value found in the *n*th patient for the *i*th QST parameter.

Although this function will always reach its maximum at 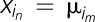, in relation to broadness of the SD, density functions can become broader or narrower. This affects the maximum value the density function can reach. To control for these more or less broad functions, we normalized the formula so that a value that is equal to the mean of the phenotype equals 100%, leading to
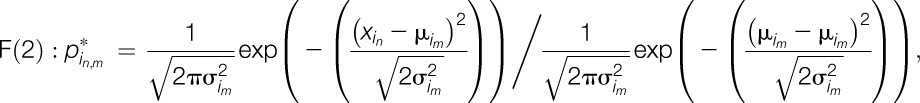


which can be simplified to
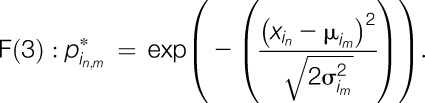


The resulting probability value ranges from 0% to 100% and can be calculated for all *i* = 13 QST parameters and *m* = 3 phenotypes. By averaging the probability over the 13 QST parameters, we quantify the similarity of the individual patient's QST profile to the mean profile of each of the 3 phenotypes.

As a simple way of categorizing patients into phenotypes, we suggest to sort each patient to the phenotype with the highest probability value:

(1) Calculate F(3) for each of the 13 QST parameters. Use μ and σ from Table 1 for SL.
(2) Average the 13 probabilities (leave out missing value from average). The resulting value is the probability for this patient to show the SL phenotype.
(3) Repeat steps 1 and 2, using μ and σ from Table 1 for thermal hyperalgesia (TH) and MH.
(4) Allocate the patient to the phenotype with the highest probability value.

**Table 1:**
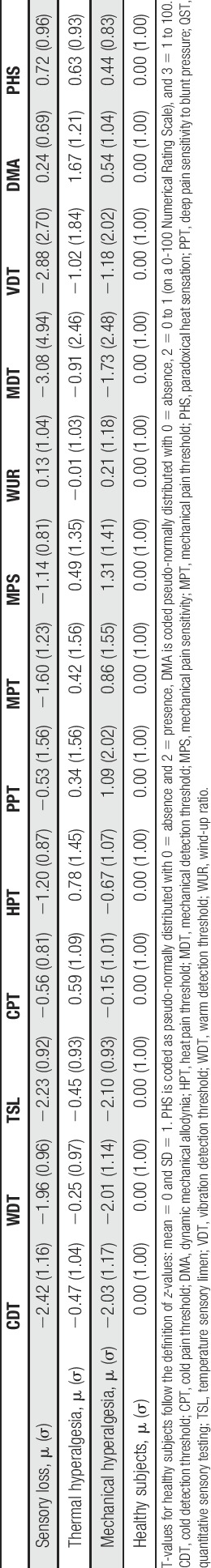
Mean QST *z*-values (μ) and SDs (σ, in brackets) for each of the 13 QST parameters separately for each of the 3 phenotypes.

The algorithm as described above was applied to the *n* = 902 patients from the original cohort^5^ to demonstrate its general sorting capacity to reproduce the original cluster allocations.

### 2.5. Simplified phenotyping

As the DFNS protocol is comprehensive, it might be too complex to be applied in all clinical settings and in large clinical trials. In our previous analysis, we showed that 2 parameters (WDT and MPS) explain large parts of the variance between the phenotypes. Therefore, we also calculated the accuracy of a phenotyping based on WDT and MPS in comparison to a phenotyping using the full protocol.

### 2.6. Discrimination analysis against healthy subjects

To show if and how the algorithm can discriminate patients with neuropathic pain from healthy subjects, we introduced a forth probability—not for a phenotype, but for being healthy. For this purpose, we applied the definition of QST *z*-values, to which a group of healthy subjects ideally has a *z*-value mean = 0 (μ) with a SD = 1 (σ) for each QST parameter. The original cluster patient cohort^5^ (n = 902) and n = 188 healthy subjects^21^ from the European cohort^39^ underwent a modified version of the algorithm:

(1) Calculate F(3) for each of the 13 QST parameters. Use μ and σ from Table 1 for healthy subjects.
(2) Average the 13 probabilities. The resulting value is the probability for this patient to show a healthy profile.
(3) Repeat steps 1 and 2, using μ and σ from Table 1 for SL, TH, and MH.

As this version of the algorithm does not sort each subject simply to the phenotype with the highest probability, this leaves every subject with 4 probabilities, one for each of the 3 phenotypes, and one for being healthy.

The probability of being healthy was used for a receiver operating characteristics (ROC) plot.^46^ This graphical tool for assessing discriminatory power plots the false-positive rate (1 − specificity) on the *x*-axis vs the sensitivity of detecting patients on the *y*-axis for all possible probability values of being healthy. Each step in the ROC plot represents the specificity and sensitivity of one certain percentage. To assess the overall quality of separating healthy subjects and patients via the probability for being healthy, the area under curve and its 95% CI were calculated.^8^ To define a minimum probability, at which a subject should be considered being healthy, the probability with the highest Youden Index (sensitivity minus false-positive rate^45^) was chosen.

### 2.7. Deterministic and probabilistic algorithm

To this point, we use a deterministic approach, ie, each patient is allocated to exactly one phenotype. It is, however, our belief that a patient may be allocated to more than 1 phenotype, so with the cut-off determined for healthy subjects above transferred onto patients, we can suggest 2 alternative versions of the algorithm, a deterministic one:

(1) Calculate F(3) for each of the 13 QST parameters. Use μ and σ from Table 1 for healthy subjects.
(2) Average the 13 probabilities. The resulting value is the probability for this patient to show a healthy profile.
(3) Repeat steps 1 and 2, using μ and σ from Table 1 for SL, TH, and MH.
(4) Allocate the patient to the phenotype with the highest probability value.

And a probabilistic version, where steps 1 to 3 remain identical and step 4 is exchanged with

(4) Sort the patient to all phenotypes with a probability above the value with the highest Youden Index found in the discrimination between patients and healthy subjects. If the only probability over this cut-off is for being healthy or no phenotype reaches a probability above this cut-off, the patient should be excluded.

These 2 versions were used for all analyses below and are presented alongside. The simplified version of the algorithm is the same, except in step 1, only WDT and MPS are used instead of all 13 QST parameters, as these parameters have shown to explain the largest part of variability between the 3 phenotypes in our previous analysis.^5^

### 2.8. Frequency of phenotypes across clinical entities

If a new drug would be tested for efficacy in a phenotype-stratified subgroup with neuropathic pain of any single etiology, this would only be meaningful if the said phenotype appears in a relevant frequency within this etiology. To show how frequent these phenotypes are across 3 common etiologies of neuropathic pain, we applied the algorithm to patients suffering from neuropathic pain due to diabetic polyneuropathy, peripheral nerve injury, or postherpetic neuralgia from the databases of our previous studies.^5,9,10,29,36^

### 2.9. Sample size recommendations

On the basis of the frequencies found in the clinical entities, we calculated the size of a group of patients who need to be screened with either full or simplified phenotyping to find a subpopulation large enough to perform a trial that still reaches a power of 80% for an effect size of 0.3, 0.5, and 0.7 at an alpha level of 0.05, for a crossover and parallel design. The sample sizes presented in this article are examples, and we encourage all readers to tailor them to their individual needs. We recommend the usage of free software G*Power,^15^ but many other statistical packages provide similar tools. The following information is required before starting: alpha level (usually 0.05), power (usually 0.8, 0.9, or 0.95), test family (usually *t* test for independent (parallel design) or dependent (crossover design) mean, or chi-squared for dichotomous outcome), and the estimated effect size in the phenotype of interest. Effect sizes are related to a mean treatment effect and SD between treatment response, eg, a mean effect of 2 on a 0 to 10 NRS scale with an SD of 4 corresponds to an effect size of 0.5, a mean effect of 3.5 with an SD of 5 corresponds to an effect size of 0.7, and a mean effect of 1 with an SD of 3 corresponds to an effect size of 0.3, and many other combinations are possible. With this information, the size of the subgroup of patients with the phenotype of interest that needs to be included can be calculated. To determine the size of the overall population which needs to be screened to find a subgroup of the calculated size, divide the subgroup size by the frequency of the phenotype in the etiology of interest as presented in the results section, in regard to the algorithm used (deterministic or probabilistic) and the phenotyping protocol (full or simplified).

## 3. Results

### 3.1. Sorting algorithm

Individual allocation replicates the original cluster analysis^5^ in 81% of the cases for the complete QST protocol using 13 parameters and in 76% of the cases using simplified phenotyping. Cohen kappa coefficient of agreement (scale: 0 = random classification, 1 = perfect agreement between methods) was 0.72 (95% CI: 0.57-0.87) for the complete protocol and 0.63 (95% CI: 0.48-0.78) for simplified phenotyping, both values may be categorized as “good,” although no universal guideline for interpreting Cohen kappa exists.^19^ Most common shifts were former SL or TH to MH (14 and 17%, respectively), and least common shift was former SL to TH (<1%). Patient shift between phenotypes is shown in Table 2.

**Table 2:**
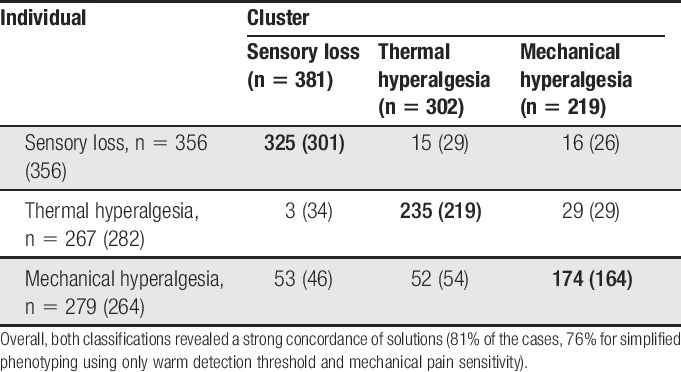
Cross-tabulation of dominant phenotype identified using cluster analysis vs the proposed new, individualized algorithm (rows) for full and simplified phenotyping (in brackets).

### 3.2. Discrimination analysis against healthy subjects

**Figure 1.**
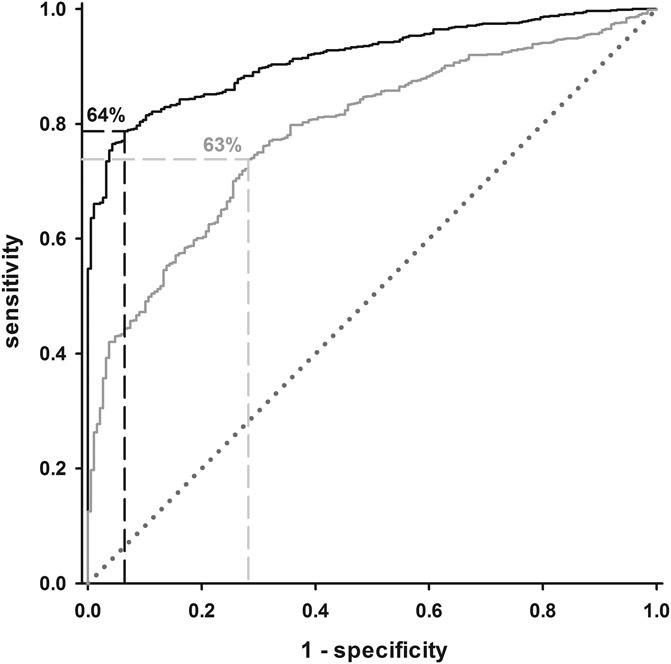
Receiver Operating Characteristic analysis of the discriminatory power of the proposed algorithm to separate between patients with neuropathic pain and healthy subjects. Black line: full sensory testing, gray line: reduced protocol, using only warm detection threshold and mechanical pain sensitivity. The green dotted diagonal line indicates random classification (“coin flipping”). The area marked by dashed lines indicates the optimum ratio of sensitivity and specificity at 64% for probability for being healthy for full phenotyping (reduced phenotyping: 63%).

**Figure 2.**
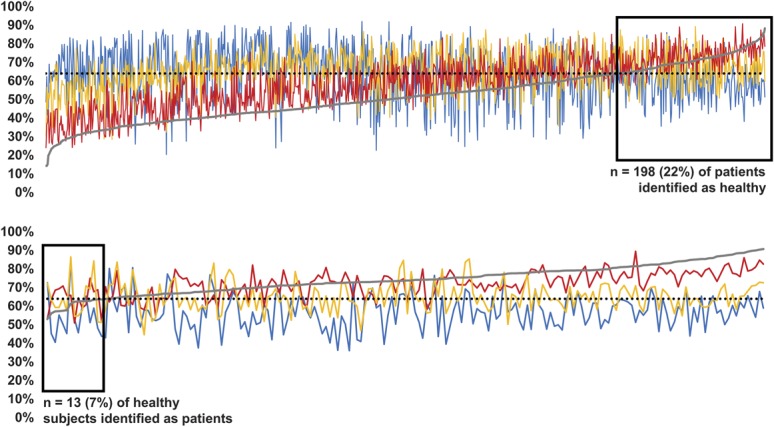
Sensory phenotype probabilities and probability of being healthy for (A) n = 902 patients with neuropathic pain and (B) n = 188 healthy subjects. Gray line: probability for being healthy, blue line: sensory loss, red line: thermal hyperalgesia, yellow line: mechanical hyperalgesia. Subjects on the *x*-axis are sorted by their individual probability of being healthy. Dotted line: a phenotype with a probability over 64% should be considered relevant in the individual patient. Thirteen healthy subjects (7%) did not reach this criterion, 198 patients (22%) had profiles consistent with being normal.

The ROC-area under curve value (scale: 0.5-1, 0.5: no discriminatory power, 1: perfect discrimination) for separating patients with neuropathic pain and healthy subjects using the probability for being healthy was found to be 0.915 (95% CI: 0.898-0.932), indicating high discriminatory power (Fig. 1). For simplified phenotyping, discriminatory power was significantly lower (0.785, 0.753-0.815). The Youden Index was found to be the highest at a probability of 64%—ie, each subject with a probability value below 64% should be considered as a patient, and when above 64% as being healthy. Although this classification is valid in 94% of healthy subjects, sensitivity in detecting patients is 78% (ie, 22% of patients with neuropathic pain have a sensory profile with a probability for being healthy above 64%). Individual probabilities for each phenotype for patients and healthy subjects are plotted in Figure 2. For simplified phenotyping, the highest Youden Index was found at a very similar value of 63% with similar sensitivity (74%) but very reduced specificity (72%). Because of the high similarity of cut-offs, 64% was used for both full protocol and simplified phenotyping.

### 3.3. Frequency of phenotypes across clinical entities

**Figure 3.**
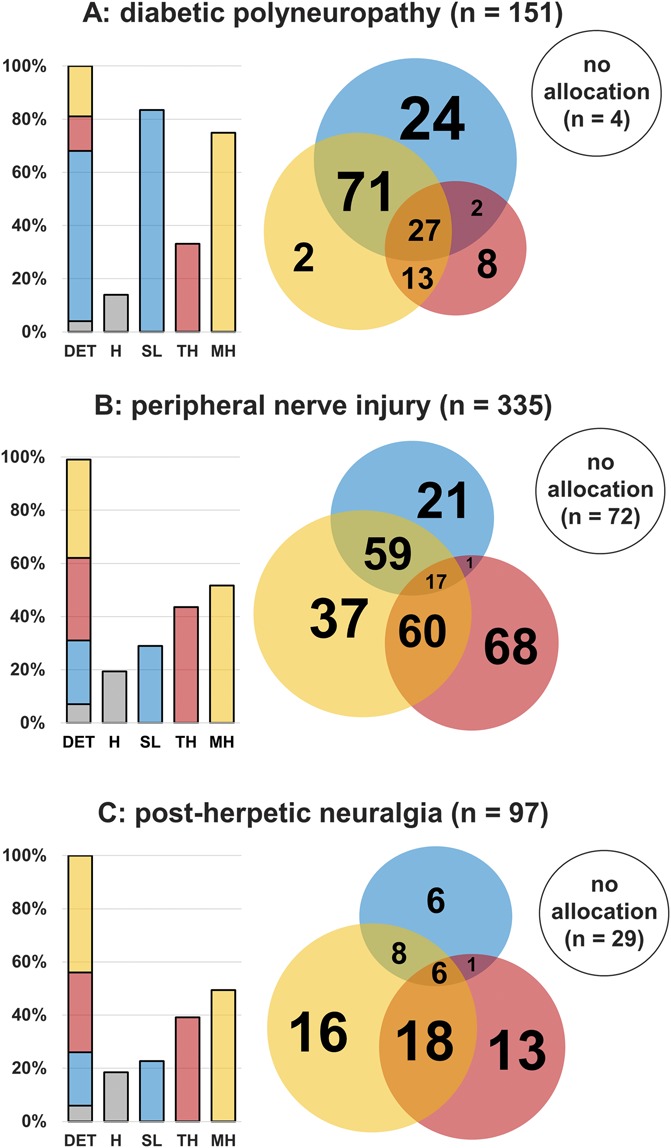
Sensory phenotype frequency and overlap between phenotypes for (A) diabetic polyneuropathy, (B) peripheral nerve injury, and (C) postherpetic neuralgia. Gray: healthy (H), blue: sensory loss (SL), red: thermal hyperalgesia (TH), yellow: mechanical hyperalgesia (MH). First bar (DET): deterministic algorithm (adds to 100%), 3 subsequent bars: probabilistic approach (a patient may be allocated to more than one phenotype, percentages are not additive). Bars are to scale, sizes of the circles in Venn diagrams and their overlaps are illustrative, not to scale.

From the databases of the DFNS, IMI Europain, Neuropain, and the Pain in Neuropathy Study (PiNS), a total of 151 patients with painful diabetic polyneuropathy, 335 patients with painful peripheral nerve injury, and 97 patients with postherpetic neuralgia who had been part of previous analyses^5,9,10,29,36^ underwent both deterministic and probabilistic phenotyping (see Table 3 for patients' basic characteristics). Frequencies of phenotypes are presented in Table 4, and frequency and overlap between phenotypes for the full protocol and for each clinical entity are displayed in Venn and bar diagrams in Figure 3.

**Table 3:**
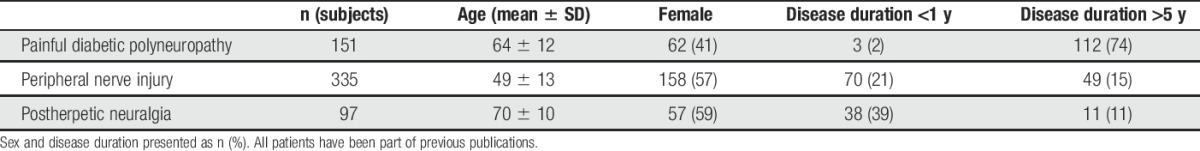
Patient characteristics separately for diabetic polyneuropathy, peripheral nerve injury, and postherpetic neuralgia.

**Table 4:**
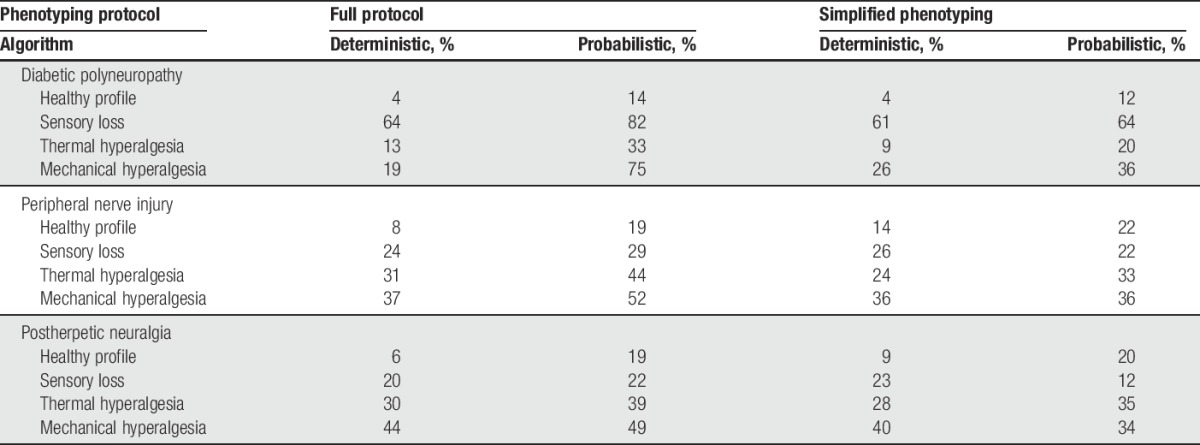
Frequency of each phenotype in diabetic polyneuropathy, peripheral nerve injury, and postherpetic neuralgia, separately for the deterministic and probabilistic algorithm, and for full and simplified phenotyping.

### 3.4. Overlap in the probabilistic algorithm

Of the diabetic polyneuropathy cohort, 4 patients (3%) were neither sorted to any phenotype nor healthy and had to be excluded. Twenty-seven patients (18%) were consistent with all 3 phenotypes and 86 (57%) with 2 phenotypes. In peripheral nerve injury, 70 (21%) patients were not assigned to any phenotype and 2 (<1%) only to the healthy profile; these patients were all excluded. Sixty-three (19%) patients were assigned to the healthy profile and at least 1 additional phenotype. Seventeen patients (5%) were allocated to all 3 phenotypes, 120 (36%) to 2 phenotypes. In postherpetic neuralgia, 29 (30%) patients were not possible to be assigned to any phenotype; these patients were excluded. Six patients (6%) were consistent with all 3 phenotypes, 27 (28%) with 2 phenotypes.

### 3.5. Accuracy of simplified phenotyping

Overall, 57% of patients with diabetic polyneuropathy, 62% of patients with peripheral nerve injury, and 58% of patients with postherpetic neuralgia were sorted into the same phenotype allocated when the full protocol was applied. The sensitivity of the simplified algorithm, however, is dependent on a combination of phenotype of interest and the clinical entity under study: In diabetic polyneuropathy, 74% of SL patients were correctly identified, but only 48% of patients with TH and 43% of patients with MH. In patients with peripheral nerve injury, allocation accuracy was more balanced between phenotypes (75% for SL, 60% for TH, and 64% for MH). In patients with postherpetic neuralgia, sensitivity was very low for SL (24%), and better for thermal (76%) and MH (56%). Consequently, low sensitivity of the simplified algorithm is linked to low frequency of certain phenotypes (especially TH in diabetic polyneuropathy and SL in postherpetic neuralgia), affecting the sample size recommendations (Table 5).

**Table 5:**
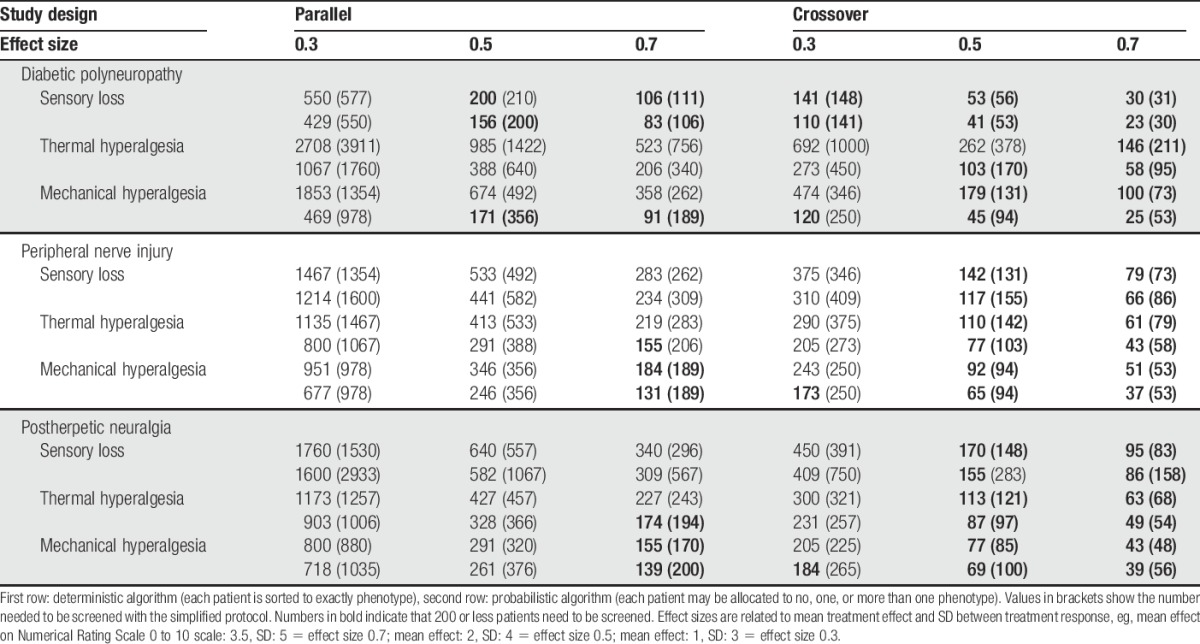
Number of patients who need to be screened to find a subpopulation with a given phenotype large enough to conduct a study with a power of 80% with an alpha-level of 0.05 and a given effect size.

## 4. Discussion

In a commentary to our findings of 3 distinct sensory phenotypes in patients suffering from neuropathic pain, we were asked to develop an algorithm to allocate individual patients to said phenotypes.^11^ This work can be considered as a response to this request. We have developed an algorithm that enables individual allocation of patients to one or more sensory phenotypes, and, further, can separate patients from healthy subjects with a very high specificity of 94% and a sensitivity of 78%. All phenotypes are present in diabetic polyneuropathy, peripheral nerve injury, and postherpetic neuralgia in reasonable frequencies, resulting in acceptable estimated sample sizes for phenotype-stratified trials, especially if crossover-designed studies are planned. The algorithm will be implemented in the next update of QST managing software eQUISTA (distributed by StatConsult, Magdeburg, Germany), but the algorithm itself is free to use.

### 4.1. Phenotype stratification by sensory profiles

A mechanistic classification of neuropathic pain has been under debate for over 25 years.^4,6,12,16,41^ Although a series of studies showed that a post hoc responder analysis can reveal phenotypes that are important to predict treatment response,^1,2,24,30,32,35,42,43^ the first phenotype-stratified, randomized, placebo-controlled trials have been published only recently.^9,10^ In these studies, oxcarbazepine showed a superior effect over placebo in a subgroup with “irritable nociceptors,” a group with a sensory profile very similar to the TH phenotype in this study. By contrast, for topical lidocaine no group difference could be demonstrated.

The main problem with the definition of “irritable nociceptors” based on abnormal QST values (ie, only values outside the 95% CI for healthy subjects are considered) and loss and gain of functions patterns^29,33^ is that it is based on a statistically sound, but conservative approach with (comparably) low sensitivity. For instance in diabetic polyneuropathy, the “irritable nociceptor” phenotype is virtually absent.^36^

By contrast, the approach taken in this study does not rely on abnormal QST values outside the 95% CI, but focuses on similarity of the entire sensory profile to cluster centroids instead. An appealing advantage of this dynamic method can be seen in Figure 3A. The TH phenotype, which is similar to the “irritable nociceptor” and may have similar underlying mechanisms of pain generation, is found to be a prominent phenotype in a reasonable subgroup of roughly one-third of the patients with neuropathic pain due to diabetic polyneuropathy.

### 4.2. Effort of stratifying populations

To use stratification into subgroups in clinical trials, a large patient population must be screened beforehand with QST to yield a smaller final stratified study population. Thus, a solid sample size calculation of the number of patients necessary to screen is a prerequisite for a stratified study. In Table 5, we present sample size numbers for screening of populations for painful diabetic polyneuropathy, painful peripheral nerve injury and postherpetic neuralgia in relation to estimated effect size (0.3 vs 0.5 vs 0.7) and study design (parallel vs crossover). Crossover sample sizes are overall “realistic” numbers–across phenotypes and clinical etiology based-entities, and are recommended for phase 2 trials. If a parallel study design is intended for phase 3 trials, however, phenotype stratification may only be possible if a high effect size (eg, 0.7) is anticipated.

### 4.3. Similarity to experimentally studied mechanisms and stratification recommendations

As discussed en detail,^5^ the phenotypes described in this study resemble sensory phenotypes that can be experimentally induced in healthy subjects. The SL phenotype is similar to previously described “deafferentation” or “painful hypoesthesia” subgroups^6,16,38^ and shows elements that can be induced by a compression nerve block.^20,44^ Edwards et al.^13^ have shown that patients with higher heat pain threshold–like these patients present–show an improved response to opioid treatment. So, a study of a centrally acting drug, eg, antidepressant or opioid, could be suggested to be stratified for this subgroup.^5,7^

The sensory profile of the TH phenotype resembles UVB burn lesion^22^ and the previously described “irritable nociceptor”.^10,16^ We suggest that sodium channel blockers would be most effective for patients with this phenotype,^5^ supported by the findings of Demant et al.^10^

The MH phenotype shows similarities to the profile induced by high-frequency electrical stimulation of the skin^26^ and the previously described “neurogenic hyperalgesia” and “central sensitization”.^6,16^ A trial investigating a calcium channel α_2_δ subunit inhibitor (coll.: gabapentinoid) or *N*-methyl-*D*-aspartate antagonist might target this phenotype,^5^ as indicated by a post hoc analysis of patients suffering from HIV-related painful neuropathy treated with pregabalin.^35^

### 4.4. Limitations

It should be emphasized that the comparison to experimentally induced mechanisms above is anecdotal rather than comprehensive.^25,27^ Finding commonalities between these clinical QST profiles and the QST profiles induced by the various surrogate models for neuropathic pain will be an important upcoming task of its own.

The sample size calculations in Table 5 show both advantage and disadvantage of a QST-based phenotype stratification for clinical trials. A novel drug that is aiming at a phenotype that is only present in a fifth of the population will never show an effect superior to placebo in a nonstratified population. However, many patients must be screened to identify an eligible subpopulation, and screening with QST needs substantial training to be reliable and should be done by certified centers. Furthermore, some QST parameters are mechanistically linked and therefore probably intercorrelated (eg, CDT or WDT and thermal sensory limen). In the presented algorithm, these domains may be slightly overweighted. Although beyond the scope of this article, a factorial analysis of the QST protocol is one of the upcoming tasks to show the importance and meaning of each parameter in relation to the full protocol. In the long run, both for large trials and daily clinical practice, an approximation via a simple bed-side testing protocol would be highly valuable.

Although the presented algorithm offers a criterion for excluding healthy subjects, this should be considered within the clinical context. We decided on a rather conservative criterion with high specificity. It has to be noted that patients eligible for clinical trials are usually screened beforehand, have been shown to have a lesion or disease, and have spontaneous pain—a sensory profile that resembles healthy subjects does not necessarily exclude a patient from a trial. Confirming neuropathic pain relies on a history of a relevant neurological lesion or disease, anatomically plausible pain distribution and sensory signs, and finally on diagnostic tests confirming the lesion or disease.^18^ Our algorithm assesses sensory signs, but only on an averaged level: eg, a strongly decreased vibration or thermal detection in an otherwise normal profile would be considered a negative sensory sign, but might still result in a high averaged probability of being healthy in this algorithm.

We present 2 methods of sorting patients to phenotypes, but we do not recommend one or the other in general, because we think both have advantages and disadvantages. The deterministic approach, sorting each patient to exactly one phenotype, ignores that multiple pathomechanisms may be present in a patient, and that these mechanisms may overlay each other and result in a sensory phenotype that cannot easily be allocated to one phenotype over the other. The probabilistic approach, however, holds its own challenges: Although the overlap between phenotypes is reasonable for peripheral nerve injury and postherpetic neuralgia, patients with diabetic polyneuropathy tend to present more than 1 phenotype with substantial probability. This effect, probably caused by the overwhelming frequency of loss symptoms in these patients, may dilute especially the MH phenotype. When screening for these phenotypes in patients with diabetic polyneuropathy, this limitation should be considered by rather using the deterministic algorithm. For peripheral nerve injury and post herpetic neuralgia, a notable part of the patients (21% and 30%, respectively) is not sorted to any phenotype in the probabilistic algorithm and therefore excluded from the analysis. Although this is acceptable for phenotype-stratified trials, it becomes a problem if the algorithm would be used for designing individual patients' treatment strategy in the future. Again, the deterministic approach might be favorable in this case.

Although this analysis focuses on trial design, it is our belief that this or a similar approach will become important in guiding individual patients' treatment in the future. This topic is heavily under debate (and there is dissent even within the author group of this article). Although we agree that at the moment we cannot present a solution to design individualized treatment based on sensory phenotypes, this analysis along with others may pave the way towards individualized pain treatment of patients with specific sensory phenotypes with future medicines.^5,7,10,30,41^

## 5. Conclusions

In summary, we present an algorithm that can be used for stratification of patients suffering from peripheral neuropathic pain in clinical trials and may in the future indicate individual patients' optimal treatment strategies. Although all 3 phenotypes are present in diabetic polyneuropathy, peripheral nerve injury, and postherpetic neuralgia, frequencies differ, which should affect the number of patients screened for clinical trials. As a result of our previous analysis,^5^ the European Medicines Agency's (EMA) committee for medicinal products for human use recommends the phenotype stratification presented here for determining eligible sensory phenotypes of patients in exploratory trials on neuropathic pain, as also incorporated in the new EMA guideline for clinical development of new treatments for pain.^14^ We encourage validation of this concept by applying it in prospectively phenotype-stratified trials on peripheral neuropathic pain.

## Conflict of interest statement

The authors have no financial or other relationships that might lead to a conflict of interest.

J. Vollert, C. Maier, R.-D. Treede, R. Baron contributed equally.

## Supplemental media

Video content associated with this article can be found online at http://links.lww.com/PAIN/A410.

## References

[1] Attal N de Andrade DC Adam F Ranoux D Teixeira MJ Galhardoni R Raicher I Üçeyler N Sommer C Bouhassira D Safety and efficacy of repeated injections of botulinum toxin A in peripheral neuropathic pain (BOTNEP): a randomised, double-blind, placebo-controlled trial. Lancet Neurol 2016;15:555–65. 26947719 10.1016/S1474-4422(16)00017-X

[2] Attal N Rouaud J Brasseur L Chauvin M Bouhassira D Systemic lidocaine in pain due to peripheral nerve injury and predictors of response. Neurology 2004;62:218–25. 14745057 10.1212/01.wnl.0000103237.62009.77

[3] Backonja MM Attal N Baron R Bouhassira D Drangholt M Dyck PJ Edwards RR Freeman R Gracely R Haanpaa MH Hansson P Hatem SM Krumova EK Jensen TS Maier C Mick G Rice AS Rolke R Treede RD Serra J Toelle T Tugnoli V Walk D Walalce MS Ware M Yarnitsky D Ziegler D Value of quantitative sensory testing in neurological and pain disorders: NeuPSIG consensus. PAIN 2013;154:1807–19. 23742795 10.1016/j.pain.2013.05.047

[4] Baron R Förster M Binder A Subgrouping of patients with neuropathic pain according to pain-related sensory abnormalities: a first step to a stratified treatment approach. Lancet Neurol 2012;11:999–1005. 23079556 10.1016/S1474-4422(12)70189-8

[5] Baron R Maier C Attal N Binder A Bouhassira D Cruccu G Finnerup NB Haanpaa M Hansson P Hullemann P Jensen TS Freynhagen R Kennedy JD Magerl W Mainka T Reimer M Rice ASC Segerdahl M Serra J Sindrup S Sommer C Tolle T Vollert J Treede RD Peripheral Neuropathic Pain: a mechanism-related organizing principle based on sensory profiles. PAIN 2017;158:261–72. 27893485 10.1097/j.pain.0000000000000753 PMC5266425

[6] Baumgartner U Magerl W Klein T Hopf HC Treede RD Neurogenic hyperalgesia versus painful hypoalgesia: two distinct mechanisms of neuropathic pain. PAIN 2002;96:141–51. 11932070 10.1016/s0304-3959(01)00438-9

[7] Bouhassira D Attal N Translational neuropathic pain research: a clinical perspective. Neuroscience 2016;338:27–35. 26995083 10.1016/j.neuroscience.2016.03.029

[8] DeLong ER DeLong DM Clarke-Pearson DL Comparing the areas under two or more correlated receiver operating characteristic curves: a nonparametric approach. Biometrics 1988;44:837–45. 3203132

[9] Demant DT Lund K Finnerup NB Vollert J Maier C Segerdahl MS Jensen TS Sindrup SH Pain relief with lidocaine 5% patch in localized peripheral neuropathic pain in relation to pain phenotype: a randomised, double-blind, and placebo-controlled, phenotype panel study. PAIN 2015;156:2234–44. 26090758 10.1097/j.pain.0000000000000266

[10] Demant DT Lund K Vollert J Maier C Segerdahl M Finnerup NB Jensen TS Sindrup SH The effect of oxcarbazepine in peripheral neuropathic pain depends on pain phenotype: a randomised, double-blind, placebo-controlled phenotype-stratified study. PAIN 2014;155:2263–73. 25139589 10.1016/j.pain.2014.08.014

[11] Dworkin RH Edwards RR Phenotypes and treatment response. PAIN 2017:158;187–89. 28092645 10.1097/j.pain.0000000000000771

[12] Edwards RR Dworkin RH Turk DC Angst MS Dionne R Freeman R Hansson P Haroutounian S Arendt-Nielsen L Attal N Baron R Brell J Bujanover S Burke LB Carr D Chappell AS Cowan P Etropolski M Fillingim RB Gewandter JS Katz NP Kopecky EA Markman JD Nomikos G Porter L Rappaport BA Rice ASC Scavone JM Scholz J Simon LS Smith SM Tobias J Tockarshewsky T Veasley C Versavel M Wasan AD Wen W Yarnitsky D Patient phenotyping in clinical trials of chronic pain treatments: IMMPACT recommendations. PAIN 2016;157:1851–71. 27152687 10.1097/j.pain.0000000000000602 PMC5965275

[13] Edwards RR Haythornthwaite JA Tella P Max MB Raja S Basal heat pain thresholds predict opioid analgesia in patients with postherpetic neuralgia. Anesthesiology 2006;104:1243–8. 16732096 10.1097/00000542-200606000-00020

[14] European Medicines Agency. EMA/CHMP/970057/2011: guideline on the clinical development of medicinal products intended for the treatment of pain. Available at: http://www.ema.europa.eu/docs/en_GB/document_library/Scientific_guideline/2016/12/WC500219131.pdf. Accessed December 15, 2016.

[15] Faul F Erdfelder E Lang AG Buchner A G*Power 3: a flexible statistical power analysis program for the social, behavioral, and biomedical sciences. Behav Res Methods 2007;39:175–91. 17695343 10.3758/bf03193146

[16] Fields HL Rowbotham M Baron R Postherpetic neuralgia: irritable nociceptors and deafferentation. Neurobiol Dis 1998;5:209–27. 9848092 10.1006/nbdi.1998.0204

[17] Finnerup NB Attal N Haroutounian S McNicol E Baron R Dworkin RH Gilron I Haanpaa M Hansson P Jensen TS Kamerman PR Lund K Moore A Raja SN Rice ASC Rowbotham M Sena E Siddall P Smith BH Wallace M Pharmacotherapy for neuropathic pain in adults: a systematic review and meta-analysis. Lancet Neurol 2015;14:162–73. 25575710 10.1016/S1474-4422(14)70251-0 PMC4493167

[18] Finnerup NB Haroutounian S Kamerman P Baron R Bennett DLH Bouhassira D Cruccu G Freeman R Hansson P Nurmikko T Raja SN Rice ASC Serra J Smith BH Treede RD Jensen TS Neuropathic pain: an updated grading system for research and clinical practice. PAIN 2016;157:1599–606. 27115670 10.1097/j.pain.0000000000000492 PMC4949003

[19] Fleiss JL Statistical methods for rates and proportions. New York: Wiley, 1973.

[20] Fruhstorfer H Thermal sensibility changes during ischemic nerve block. PAIN 1984;20:355–61. 6522070 10.1016/0304-3959(84)90112-X

[21] Gierthmuhlen J Enax-Krumova EK Attal N Bouhassira D Cruccu G Finnerup NB Haanpaa M Hansson P Jensen TS Freynhagen R Kennedy JD Mainka T Rice ASC Segerdahl M Sindrup SH Serra J Tolle T Treede R-D Baron R Maier C Who is healthy? Aspects to consider when including healthy volunteers in QST–based studies-a consensus statement by the EUROPAIN and NEUROPAIN consortia. PAIN 2015;156:2203–11. 26075963 10.1097/j.pain.0000000000000227

[22] Gustorff B Sycha T Lieba-Samal D Rolke R Treede RD Magerl W The pattern and time course of somatosensory changes in the human UVB sunburn model reveal the presence of peripheral and central sensitization. PAIN 2013;154:586–97. 23419598 10.1016/j.pain.2012.12.020

[23] Katz J Finnerup NB Dworkin RH Clinical trial outcome in neuropathic pain: relationship to study characteristics. Neurology 2008;70:263–72. 17914067 10.1212/01.WNL.0000275528.01263.6c

[24] Katz NP Mou J Paillard FC Turnbull B Trudeau J Stoker M Predictors of response in patients with postherpetic neuralgia and HIV-associated neuropathy treated with the 8% capsaicin patch (Qutenza). Clin J Pain 2015;31:859–66. 25503598 10.1097/AJP.0000000000000186

[25] Klein T Magerl W Rolke R Treede RD Human surrogate models of neuropathic pain. PAIN 2005;115:227–33. 15876495 10.1016/j.pain.2005.03.021

[26] Lang S Klein T Magerl W Treede RD Modality-specific sensory changes in humans after the induction of long-term potentiation (LTP) in cutaneous nociceptive pathways. PAIN 2007;128:254–63. 17123732 10.1016/j.pain.2006.09.026

[27] Lotsch J Oertel BG Ultsch A Human models of pain for the prediction of clinical analgesia. PAIN 2014;155:2014–21. 25020003 10.1016/j.pain.2014.07.003

[28] Magerl W Krumova EK Baron R Tolle T Treede RD Maier C Reference data for quantitative sensory testing (QST): refined stratification for age and a novel method for statistical comparison of group data. PAIN 2010;151:598–605. 20965658 10.1016/j.pain.2010.07.026

[29] Maier C Baron R Tolle TR Binder A Birbaumer N Birklein F Gierthmuhlen J Flor H Geber C Huge V Krumova EK Landwehrmeyer GB Magerl W Maihofner C Richter H Rolke R Scherens A Schwarz A Sommer C Tronnier V Uceyler N Valet M Wasner G Treede RD Quantitative sensory testing in the German Research Network on Neuropathic Pain (DFNS): somatosensory abnormalities in 1236 patients with different neuropathic pain syndromes. PAIN 2010;150:439–50. 20627413 10.1016/j.pain.2010.05.002

[30] Mainka T Malewicz NM Baron R Enax-Krumova EK Treede RD Maier C Presence of hyperalgesia predicts analgesic efficacy of topically applied capsaicin 8% in patients with peripheral neuropathic pain. Eur J Pain 2016;20:116–29. 25854794 10.1002/ejp.703

[31] Pfau DB Krumova EK Treede RD Baron R Toelle T Birklein F Eich W Geber C Gerhardt A Weiss T Magerl W Maier C Quantitative sensory testing in the German Research Network on Neuropathic Pain (DFNS): reference data for the trunk and application in patients with chronic postherpetic neuralgia. PAIN 2014;155:1002–15. 24525274 10.1016/j.pain.2014.02.004

[32] Reimer M Hullemann P Hukauf M Keller T Binder A Gierthmuhlen J Baron R Prediction of response to tapentadol in chronic low back pain. Eur J Pain 2017;21:322–33. 27510567 10.1002/ejp.926 PMC5248647

[33] Rolke R Baron R Maier C Tolle TR Treede RD Beyer A Binder A Birbaumer N Birklein F Botefur IC Braune S Flor H Huge V Klug R Landwehrmeyer GB Magerl W Maihofner C Rolko C Schaub C Scherens A Sprenger T Valet M Wasserka B Quantitative sensory testing in the German Research Network on Neuropathic Pain (DFNS): standardized protocol and reference values. PAIN 2006;123:231–43. 16697110 10.1016/j.pain.2006.01.041

[34] Rolke R Magerl W Campbell KA Schalber C Caspari S Birklein F Treede RD Quantitative sensory testing: a comprehensive protocol for clinical trials. Eur J Pain 2006;10:77–88. 16291301 10.1016/j.ejpain.2005.02.003

[35] Simpson DM Schifitto G Clifford DB Murphy TK Durso-de Cruz E Glue P Whalen E Emir B Scott GN Freeman R Pregabalin for painful HIV neuropathy: a randomized, double-blind, placebo-controlled trial. Neurology 2010;74:413–20. 20124207 10.1212/WNL.0b013e3181ccc6ef PMC2816006

[36] Themistocleous AC Ramirez JD Shillo PR Lees JG Selvarajah D Orengo C Tesfaye S Rice ASC Bennett DLH The Pain in Neuropathy Study (PiNS): a cross-sectional observational study determining the somatosensory phenotype of painful and painless diabetic neuropathy. PAIN 2016;157:1132–45. 27088890 10.1097/j.pain.0000000000000491 PMC4834814

[37] Treede RD Jensen TS Campbell JN Cruccu G Dostrovsky JO Griffin JW Hansson P Hughes R Nurmikko T Serra J Neuropathic pain: redefinition and a grading system for clinical and research purposes. Neurology 2008;70:1630–5. 18003941 10.1212/01.wnl.0000282763.29778.59

[38] Truini A Padua L Biasiotta A Caliandro P Pazzaglia C Galeotti F Inghilleri M Cruccu G Differential involvement of A-delta and A-beta fibres in neuropathic pain related to carpal tunnel syndrome. PAIN 2009;145:105–9. 19535205 10.1016/j.pain.2009.05.023

[39] Vollert J Attal N Baron R Freynhagen R Haanpaa M Hansson P Jensen TS Rice ASC Segerdahl M Serra J Sindrup SH Tolle TR Treede RD Maier C Quantitative sensory testing using DFNS protocol in Europe: an evaluation of heterogeneity across multiple centers in patients with peripheral neuropathic pain and healthy subjects. PAIN 2016;157:750–8. 26630440 10.1097/j.pain.0000000000000433

[40] Vollert J Mainka T Baron R Enax-Krumova EK Hullemann P Maier C Pfau DB Tolle T Treede R-D Quality assurance for Quantitative Sensory Testing laboratories: development and validation of an automated evaluation tool for the analysis of declared healthy samples. PAIN 2015;156:2423–30. 26580679 10.1097/j.pain.0000000000000300

[41] von Hehn CA Baron R Woolf CJ Deconstructing the neuropathic pain phenotype to reveal neural mechanisms. Neuron 2012;73:638–52. 22365541 10.1016/j.neuron.2012.02.008 PMC3319438

[42] Wasner G Kleinert A Binder A Schattschneider J Baron R Postherpetic neuralgia: topical lidocaine is effective in nociceptor-deprived skin. J Neurol 2005;252:677–86. 15778907 10.1007/s00415-005-0717-z

[43] Westermann A Krumova EK Pennekamp W Horch C Baron R Maier C Different underlying pain mechanisms despite identical pain characteristics: a case report of a patient with spinal cord injury. PAIN 2012;153:1537–40. 22444186 10.1016/j.pain.2012.02.031

[44] Yarnitsky D Ochoa JL Differential effect of compression-ischaemia block on warm sensation and heat-induced pain. Brain 1991;114:907–13. 2043957 10.1093/brain/114.2.907

[45] Youden WJ Index for rating diagnostic tests. Cancer 1950;3:32–5. 15405679 10.1002/1097-0142(1950)3:1<32::aid-cncr2820030106>3.0.co;2-3

[46] Zweig MH Campbell G Receiver-operating characteristic (ROC) plots: a fundamental evaluation tool in clinical medicine. Clin Chem 1993;39:561–77. 8472349

